# Idiopathic confetti-like leukoderma: A case report of a rare entity

**DOI:** 10.1097/MD.0000000000046806

**Published:** 2025-12-26

**Authors:** Kundan Kumar Yadav, Sonam Dhenga, Milan Pokhrel, Bibek Shrestha, Srijana Kumari Yadav, Sagar Bishowkarma, Ravi Kumar Yadav, Dhiraj Kumar Das

**Affiliations:** a Tribhuvan University, Institute of Medicine, Maharajgunj, Kathmandu, Nepal; b Universal College of Medical Sciences, Bhairahawa, Ranigaon.

**Keywords:** confetti-like leukoderma, idiopathic, vitilligo

## Abstract

**Rationale:**

Confetti-like leukoderma is an uncommon and intriguing hypopigmentary presentation typically associated with systemic illnesses, dermatologic disorders, or adverse reactions to therapies. Its idiopathic form is extremely rare, making such cases clinically significant and worthy of documentation.

**Patient concerns:**

A 17-year-old male presented with multiple, bilateral, symmetrical hypopigmented macules measuring 1 to 3 mm, diffusely distributed over the trunk, extremities, neck, forehead, and dorsum of the hands. He denied chemical exposures, systemic symptoms, photosensitivity, or family history of similar lesions.

**Diagnoses:**

Clinical examination and laboratory studies, including complete blood count, autoimmune markers, arsenic levels, and imaging, were normal. Differential diagnoses considered included systemic sclerosis, arsenicosis, and other hypopigmentary disorders. Histopathology showed scattered melanocytes, pigment incontinence, melanophages, and mild perivascular lymphocytic infiltrate, confirming idiopathic confetti-like leukoderma.

**Interventions:**

No therapeutic intervention was initiated. Diagnosis was established after careful exclusion of secondary causes through clinical, laboratory, and histopathological correlation.

**Outcomes:**

The patient remained clinically stable, and no progression or systemic involvement was noted. The condition was recognized as an isolated idiopathic entity with no immediate need for treatment.

**Lessons:**

This case highlights idiopathic confetti-like leukoderma as a rare but distinct clinical diagnosis. Comprehensive evaluation, exclusion of systemic associations, and histopathologic confirmation are essential. As only a handful of such idiopathic cases have been reported, further recognition and documentation are necessary to better understand this presentation.

## 1. Introduction

A number of skin disorders may present with hypopigmentation. The distinct appearance of those hypo-pigmented lesions, such as guttate, speckled, or confetti-like like and their distribution help in reaching a diagnosis or at least narrowing down differentials.^[1]^ Leukoderma punctatum, Confetti-like hypopigmentation was originally reported as a separate entity by Falabela et al in 1988.^[2]^ Confetti-like leukoderma refers to numerous asymptomatic hypo- to depigmented cutaneous macules of size 1 to 3 mm in diameter. It has been reported to appear primarily at the borders of preexisting lesions in groups and usually spares the follicular or perifollicular region. Thus far, the condition has been hardly documented in literature, and those reported are mostly in the context of either topical and/or systemic therapy or as a key cutaneous marker of scores of multiple systemic disorders. Hence, it is justifiable to report a case of this entity.^[2–6]^

In this report, we present an uncommon set of clinical and histopathological features that do not fall under the diagnostic features of any known skin disorders, where confetti leucoderma and confetti-like skin lesions are present.

## 2. Case presentation

**Figure 1.**
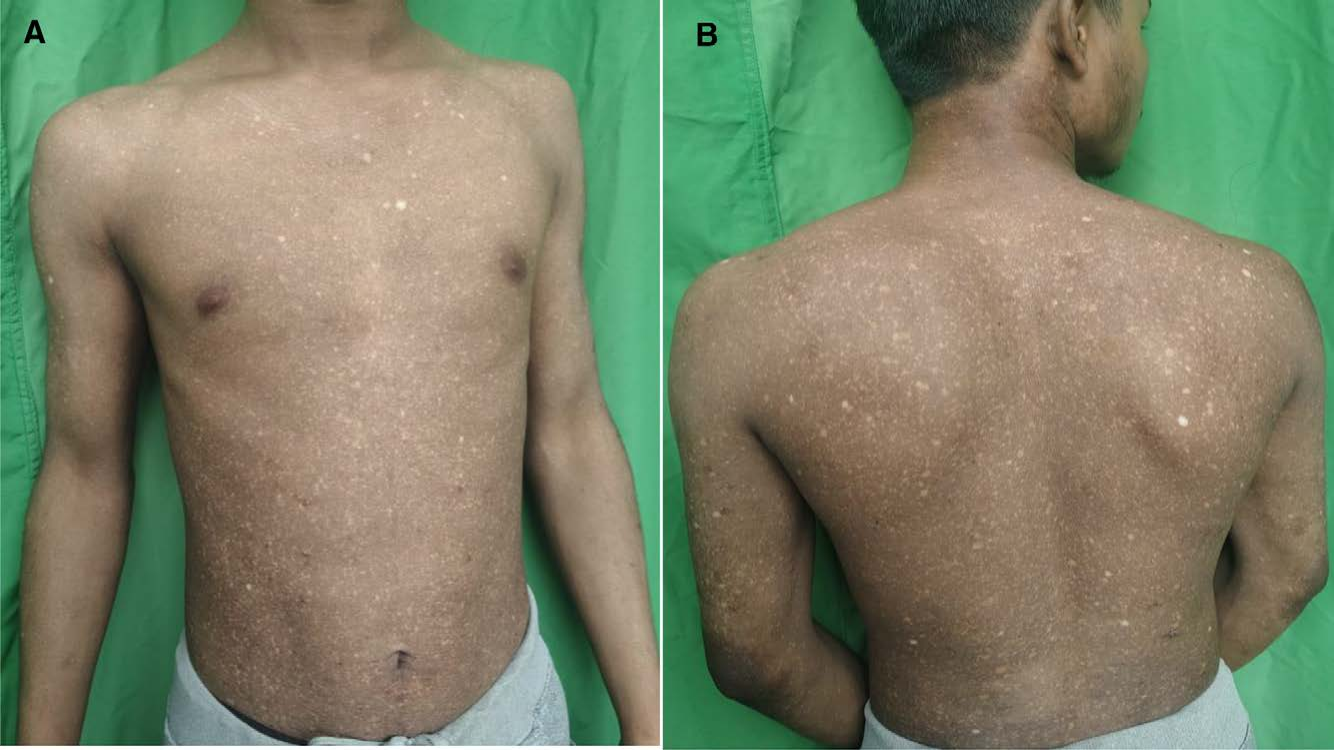
(A, B) Diffuse hypo to depigmented confetti-like macules measuring 1 to 3mm distributed over the trunk and bilateral hands.

A 17-year-old male presented with multiple small whitish lesions all over his body. The lesion was first noticed by his mother around 5 years ago on his back, then it had started progressing. History of itchiness and redness of the skin was denied. No complaints of tightening or thickening of skin were present. There was no history of joint pain or photosensitivity. There was no known exposure to any chemical or topical/systemic medication. He did not have any preexisting dermatoses. No similar cutaneous findings were noticed in his family members or village neighbors. The primary source of drinking water was Jar water. The patient had a darker skin tone. Examination revealed diffuse hypo to depigmented confetti-like macules measuring 1 mm to 3 mm over healthy-looking skin in his trunk, bilateral extremities, neck, pinnae, forehead, mandibular region, and dorsum of both hands. They were bilateral and symmetrical. Mucosa, palms, and soles were spared. Systemic examination did not reveal any abnormality. With these clinical findings, differential diagnoses of systemic sclerosis, arsenic poisoning, and idiopathic confetti-like leukoderma were made (Fig. 1A,B).

**Figure 2.**
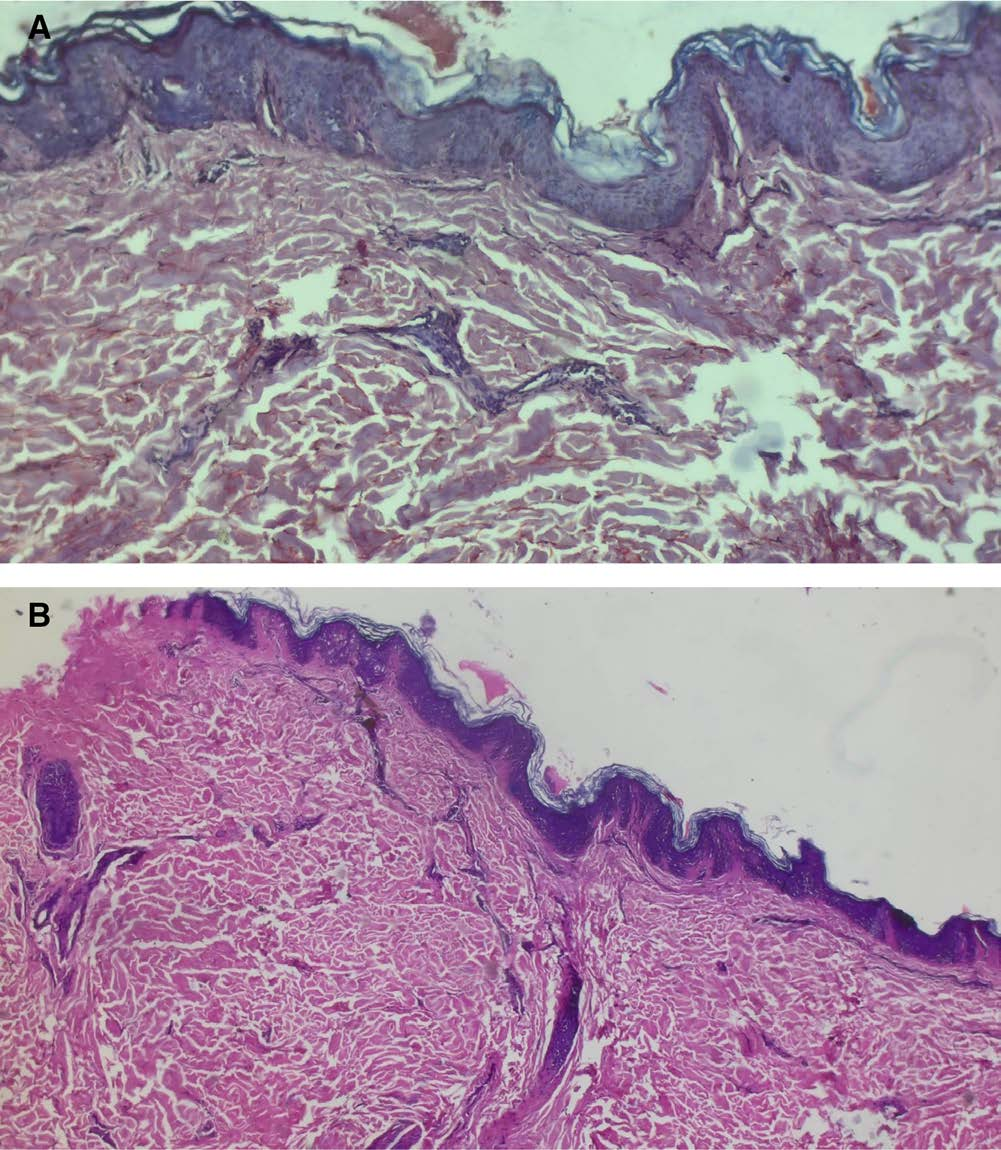
(A) Histopathological image showing adequate melanin pigmentation of basal keratinocytes in some areas, while it was reduced in other areas of epidermis with scattered melanocytes. Superficial dermis showed pigment incontinence, melanophages, and mild perivascular lymphocytic infiltrate. (B) Congo red stain revealing absence of amyloid.

However, blood investigations like complete blood count, erythrocyte sedimentation rate, antinuclear antibody, kidney function tests, liver function tests, urine analysis, and chest X-ray did not show any abnormal findings. Tests to see arsenic levels in hair and nail were done, and it was unremarkable. Ten percent potassium hydroxide mount prepared from the scrapings was negative for any spores or hyphae. Incisional Biopsy of the representative lesion was done. Epidermis showed adequate melanin pigmentation of basal keratinocytes in some areas, while it was reduced in other areas. There were scattered melanocytes. Superficial dermis showed pigment incontinence, melanophages, and mild perivascular lymphocytic infiltrate. Subcutis showed thick fibrous septa with collagen deposition. The size of the adipocyte lobule was decreased (Fig. 2A). Congo red stain did not reveal any amyloid (Fig. 2B). Thus, by excluding all other differentials on the basis of clinical, laboratory, and histopathological findings, the diagnosis of idiopathic confetti-like leukoderma was made.

## 3. Discussion

Leukoderma punctatum, or confetti-like hypopigmentation, was first described as a consequence of phototherapy for vitiligo and psoriasis.^[2]^ Since then, it has been associated with a variety of other skin disorders, including chemical leucoderma, tuberous sclerosis, pityriasis versicolor, idiopathic guttate hypomelanosis, familial white lentiginosis, Darier disease, Cole disease, multiple endocrine neoplasia type 1, Nevus achromicus, and piebaldism.^[1,2,7]^ This manifestation may easily be mistaken for other pigmentary patterns, such as raindrop pigmentation in arsenicosis or salt-and-pepper pigmentation in systemic sclerosis.^[8,9]^ Careful clinical examination supported by relevant investigations is necessary to exclude these possibilities.

In vitiligo, confetti-like depigmentation has been considered both a negative prognostic marker and an adverse effect of phototherapy. Ultrastructural studies have shown evidence of keratinocyte and melanocyte damage, characterized by intracellular edema and intracytoplasmic vacuolar degeneration.^[2]^ These lesions are often seen alongside active vitiligo patches such as trichrome or hypochromic lesions.^[5]^ Histopathological examination typically reveals a lymphocytic infiltrate dominated by CD8 + T cells at the dermoepidermal junction, which directly contributes to melanocyte destruction.^[4,5]^

Confetti-like macules are also a hallmark of chemical leukoderma. They usually develop at the site of repeated exposure to depigmenting agents such as hydroquinone but may later appear at distant body sites as well.^[3,7]^ The extent and distribution of the lesions often depend on the timing of clinical observation, as macules may confluence with progression, and depigmentation is dose-dependent. In some cases, patients show abnormal liver function, hepatosplenomegaly, or thyroid dysfunction, and a family history of vitiligo or chemical leukoderma may be present. Importantly, spontaneous repigmentation has been reported following withdrawal of the causative agent, though this is typically slow and occurs over weeks to months.^[7]^

Systemic sclerosis is another important differential, manifesting as salt-and-pepper pigmentation, most frequently in individuals with darker skin tones and sun-exposed areas. The vitiligo-like depigmentation with perifollicular pigment retention is believed to arise from trauma, friction, thermovascular changes, photoexposure, or immune-mediated melanocyte injury.^[8,10]^ The supraclavicular and suprascapular regions are most commonly affected, though the scalp, forehead, neck, dorsal hands, and forearms are also frequently involved. These changes typically coexist with areas of cutaneous thickening and fibrosis. In contrast, arsenicosis most often presents with raindrop pigmentation, which includes mottled or finely freckled dyspigmentation, blotchy mucosal pigmentation, palmoplantar pitting, and keratosis, most prominently on the trunk and extremities. Defined by the World Health Organization as a chronic health condition due to ingestion of unsafe levels of arsenic for at least 6 months, arsenicosis is characterized by typical skin lesions, with or without systemic organ involvement.^[9,11]^

Several congenital and acquired conditions may present with similar confetti-like changes. In tuberous sclerosis, hypopigmented macules are often present from birth or early infancy, usually remaining stable throughout life.^[3,7]^ Piebaldism also appears at birth, presenting as depigmented patches often localized on the forehead with associated white hair, and these lesions do not progress over time. Nevus achromicus may also be congenital, with stable depigmented patches sometimes linked to preceding inflammation. In contrast, pityriasis versicolor presents as hypopigmented, scaly lesions, usually confined to the trunk, with characteristic fluorescence under Wood’s lamp and microscopic evidence of hyphae and spores, while pityriasis alba in children produces ill-defined, scaly patches, most often on the cheeks and upper limbs.^[7]^ Rarely, Darier disease may display hypopigmented confetti-like macules before the appearance of its more classic hyperkeratotic plaques, with histopathology demonstrating acantholytic dyskeratosis and reduced melanocytes.^[12]^ Familial white lentiginosis, more common in individuals of African descent, typically shows truncal lesions, whereas acromelanosis albo-punctata combines diffuse hyperpigmentation with punctate hypopigmented macules on the hands and feet.^[1]^ Early stages of stucco keratosis, lichen sclerosus et atrophicus, and verrucae planae juveniles may also mimic confetti-like leukoderma in their initial phases.^[6]^

Pathogenetically, confetti leukoderma is thought to occur due to alterations in melanin production, defects in melanin transfer, or direct destruction of melanocytes. Special staining has confirmed a reduction in functional melanocytes.^[6]^ In our patient, the clinical course, demographic profile, histopathological findings, and disease characteristics suggest that the confetti-like leukoderma observed does not fit into any of the previously described conditions. To date, only one similar case has been documented, making this report possibly the second of its kind in the world literature. The precise etiology and mechanisms remain uncertain, which may be explained by the extreme rarity of this entity.

## 4. Conclusion

When confetti-like leukoderma itself is an unusual skin trait, studies regarding its natural history, pathogenesis, and classification seem to be beyond consideration. It is therefore imperative to perform a critical evaluation of its fundamental clinical characteristics, histopathological features, and pertinent investigations as warranted to diagnose the entity and classify it into primary (idiopathic), secondary, and as a recognized skin marker in related systemic and/or cutaneous conditions.

## Author contributions

**Conceptualization:** Kundan Kumar Yadav, Sonam Dhenga.

**Writing – original draft:** Kundan Kumar Yadav, Sonam Dhenga, Milan Pokhrel.

**Writing – review & editing:** Kundan Kumar Yadav, Sonam Dhenga, Milan Pokhrel, Bibek Shrestha, Srijana Kumari Yadav, Sagar Bishwokarma, Ravi Kumar Yadav, Dhiraj Kumar Das.

## References

[1] Aldhalaan J Ghobara YA Alisaa A AlJasser MI. Acral speckled hypomelanosis. JAAD Case Rep. 2019;5:770–2. 31516993 10.1016/j.jdcr.2019.06.024 PMC6728832

[2] Bhatnagar A Kanwar A Parsad D Narang T De D. Confetti‐like hypopigmentation: a rare complication of common phototherapeutic modality. Acad Dermatol Venereol. 2007;21:1276–7. 10.1111/j.1468-3083.2007.02170.x 17894733

[3] Sehgal VN Sharma S Verma P Srivastava G Gupta M. Idiopathic confetti-like leukoderma with unusual presentation. Am J Dermatopathol. 2012;34:117–21. 22197857 10.1097/DAD.0b013e31821a2b76

[4] Sosa JJ Currimbhoy SD Ukoha U. Confetti-like depigmentation: a potential sign of rapidly progressing vitiligo. J Am Acad Dermatol. 2015;73:272–5. 26054430 10.1016/j.jaad.2015.05.014

[5] Van Geel N Grine L De Wispelaere P Mertens D Prinsen CAC Speeckaert R. Clinical visible signs of disease activity in vitiligo: a systematic review and meta‐analysis. J Eur Acad Dermatol Venereol. 2019;33:1667–75. 31131483 10.1111/jdv.15604

[6] Loquai C Metze D Nashan D Luger TA Bohm M. Confetti-like lesions with hyperkeratosis: a novel ultraviolet-induced hypomelanotic disorder? Br J Dermatol. 2005;153:190–3. 16029349 10.1111/j.1365-2133.2005.06634.x

[7] Bonamonte D Vestita M Romita P Filoni A Foti C Angelini G. Chemical leukoderma. Dermatitis. 2016;27:90–9. 27172302 10.1097/DER.0000000000000167

[8] Pearson DR Werth VP Pappas-Taffer L. Systemic sclerosis: Current concepts of skin and systemic manifestations. Clin Dermatol. 2018;36:459–74. 30047430 10.1016/j.clindermatol.2018.04.004

[9] Bhanja DB Sil A Sen SS Chandra A. Chronic arsenicosis. BMJ Case Rep. 2021;14:e244071. 10.1136/bcr-2021-244071 PMC8286736 34266830

[10] Gnanasuriyan R Murali S Kuruvila S. Salt-and-pepper skin pigmentation. Cleve Clin J Med. 2024;91:593–4. 39353660 10.3949/ccjm.91a.24038

[11] Das A Toshniwal A Majumdar K. Rain drop pigmentation in chronic arsenic poisoning. Pigment Int. 2020;7:61.

[12] Sun CW Grossman SK Valdes-Rodriguez R Lee JB Hsu S. Guttate leukoderma and acrokeratosis verruciformis of Hopf: a rare combination in Darier disease. Dermatol Online J. 2020;26:13030/qt5938q4rj. 32155025

